# A DFX-based iron nanochelator for cancer therapy

**DOI:** 10.3389/fbioe.2022.1078137

**Published:** 2022-11-28

**Authors:** Peng Liu, Qiang Wang, Kuan Li, Bo Bi, Ying-Fei Wen, Miao-Juan Qiu, Jing Zhao, Bin-Bin Li, Chang-Hua Zhang, Yu-Long He

**Affiliations:** ^1^ Digestive Diseases Center, The Seventh Affiliated Hospital of Sun Yat-Sen University, Sun Yat-Sen University, Shenzhen, Guangdong, China; ^2^ Guangdong Provincial Key Laboratory of Digestive Cancer Research, The Seventh Affiliated Hospital of Sun Yat-sen University, Sun Yat-Sen University, Shenzhen, Guangdong, China; ^3^ Scientific Research Center, The Seventh Affiliated Hospital of Sun Yat-Sen University, Sun Yat-Sen University, Shenzhen, Guangdong, China

**Keywords:** polylysine, deferasirox, iron, nanochelator, cancer therapy

## Abstract

Iron as an essential element, is involved in various cellular functions and maintaining cell viability, cancer cell is more dependent on iron than normal cell due to its chief characteristic of hyper-proliferation. Despite that some of the iron chelators exhibited potent and broad antitumor activity, severe systemic toxicities have limited their clinical application. Polyaminoacids, as both drug-delivery platform and therapeutic agents, have attracted great interests owing to their different medical applications and biocompatibility. Herein, we have developed a novel iron nanochelator PL-DFX, which composed of deferasirox and hyperbranched polylysine. PL-DFX has higher cytotoxicity than DFX and this effect can be partially reversed by Fe^2+^ supplementation. PL-DFX also inhibited migration and invasion of cancer cells, interfere with iron metabolism, induce phase G1/S arrest and depolarize mitochondria membrane potential. Additionally, the anti-tumor potency of PL-DFX was also supported by organoids derived from clinical specimens. In this study, DFX-based iron nanochelator has provided a promising and prospective strategy for cancer therapy *via* iron metabolism disruption.

## Introduction

Iron is fundamental for cell function involved biomolecule syntheses, respiration, metabolism and DNA replication. Cancer cell requires more iron to facilitate its proliferation and growth (Zhang, 2014; Torti et al., 2018), which has been investigated by numerous studies conducted in cell, animal model and epidemiology (Torti and Torti, 2013). A meta-analysis involved 33 studies showed that higher iron intake increased the risk of colorectal cancer (Nelson, 2001). Multiple pathway such as Wnt and JAK-STAT3 signaling has been activated in tumor development and metastasis induced by iron overload (Ebina et al., 1986; Hann et al., 1991; Brookes et al., 2008; Xue et al., 2016; Schwartz et al., 2021), while iron depletion led to suppression of tumor growth and metastasis (Torti et al., 2018).

Iron chelators, like deferoxamine (DFO) and deferasirox (DFX), which can decrease the iron level in tissue, are commonly used for the treatment of iron-overload diseases such as thalassemia (Sridharan and Sivaramakrishnan, 2018). Accumulating evidence has revealed that iron chelators have robust and broad antitumor activities (Yu et al., 2012), and also have clinical efficacy in non-neoplastic diseases (Xu et al., 2022). Deferasirox, also known as ICL670, is a kind of oral iron chelator and has been approved by FDA for clinical treatment of blood-transfusion-related iron overload (Diaz-Garcia et al., 2014). In contrast to DFO, DFX has similar or better efficacy and more favorable safety profile (Nick et al., 2002; Cappellini, 2008; Vichinsky et al., 2013). Deferasirox also demonstrated antitumor effect in oesophageal, cervical, pancreatic, lung and gastric cancer (Ford et al., 2013; Lui et al., 2013; Choi et al., 2016; Amano et al., 2020; Zhou et al., 2022). However, deferasirox, as a small molecular agent, has several severe adverse effects, the most common was nephrotoxicity and occurred in ten percent of patients who received iron chelation treatment (Gattermann et al., 2010; Diaz-Garcia et al., 2014; Kattamis, 2019). Thus, despite iron homeostasis is a promising target in different cancer models, there is a lack of efficient and safe delivery system to suppress the side effect of deferasirox and maintain its efficiency at the same time.

The rise of nanomedicine has provided new strategies for cancer therapy (He et al., 2021; Yang et al., 2022). Polyaminoacids has attracted great attention in the regards of both bioactive agents and drug carrier. Polyaminoacids was characterized by good biocompatibility, ease of modification and slow degradability (Boddu et al., 2021). Polylysine, which produced by *streptomyces albulus*, is a natural poly (amino acid) polymer composed of lysine with amino groups on the side chains (Tao et al., 2015; Boddu et al., 2021). Polylysine, as a drug carrier for cancer therapy, possesses the following advantages: (A) Polylysine can enhance the therapeutic efficacy of drugs loaded. For example, polylysine can enhance the therapeutic efficacy of drugs when polymerize it with methotrexate, (B) polylysine is rich in cations, thus can penetrate biofilms and especially interact with tumor cells commonly possessing negatively charged membranes (Vasir and Labhasetwar, 2008; Zhou et al., 2015; Narayanan et al., 2022), and (C) Polylysine is biodegradable which could prevent accumulative cytotoxicity and facilitate downstream processing. Herein, a DFX-based iron nanochelator, which was formed by deferasirox loaded hyperbranched polylysine, was designed and synthesized for cancer therapy *via* iron deficiency. PL-DFX induced dysregulation of the iron homeostasis and increased the potency of DFX in gastrointestinal tumor cells. PL-DFX also demonstrated remarkable antitumor effects in patient-derived gastric and colorectal tumor organoids. Overall, this novel iron nanochelator provides new insights for cancer therapy.

## Materials and methods

### Materials

Deferasirox was purchased from Aladdin Biotechnology (Shanghai, Chain). Cell culture medium, trypsin, penicillin-streptomycin and fetal bovine serum were purchased from Gibco (Guangzhou, China). Cell counting kit-8 (CCK8) was purchased from Yeasen (shanghai, China). Annexin V-FITC/PI apoptosis Kit, cell cycle kit, mitochondria membrane potential detection (JC-1) kit and calcein-AM were purchased from Beyotime (Shanghai, China).

### Syntheses of hyperbranched polylysine

**FIGURE 1:**
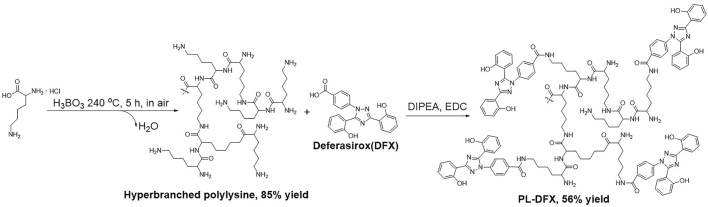
Schematic illustration of PL-DFX synthesis and fabrication.

Firstly, the hyperbranched polylysine was synthesized by following method (Figure 1): Lysine·HCl (27.40 g, 150 mmol) and KOH (8.42 g, 150 mmol) was completely stirred by mortar until well mixed. The mixture was transferred into open 1 L round bottom flask and stirred under 240°C with 3 mol% H_3_BO_3_ as catalyst. The flask was opened to allow water formed in the reaction to escape. The reaction was stopped and cooled to room temperature after 5 h. The crud product was collected by dissolving in methanol. The KCl formed during the reaction was filtered off. The methanol was evaporated and the product was dissolved in water. The aqueous solution was then lyophilized to afford polylysine solid in 85% yield.

### Syntheses of PL-DFX

The DFX loading reaction was carried out as follows (Figure 1): the hyperbranched polylysine (274.0 mg, 1.50 mmol) synthesized in the former step was dissolved in DMSO (30 ml), DIPEA (100 μl) was then added to the solution. DFX (280.0 mg, 0.75 mmol) in DMSO (20 ml) and EDC (152.2 mg, 0.080 mmol) was added. The two solution was then mixed in one flask and stirred at room temperature for 5 h. The solution was dialyzed (Spectra/pro MWCO = 1000) against acetonitrile (500 ml). The precipitate was collected and washed with acetonitrile for three times in a glass filter. The crude product was dissolved in minimum of DMSO and dialyzed against water (500 ml, three times). The product was lyophilized to appear as white to yellow solid (yield = 56%).

### Characterization of PL-DFX

^1^H NMR was recorded at room temperature on a Bruker Avance III 600 MHz nuclear magnetic resonance spectrometer; FTIR: IR spectra was recorded by using FT-IR Spectrometer Platform INVENIO; UV-Vis: UV-Vis spectra was measured by Agilent Cary UV-Vis spectormeter; Lyophilized: Lypholization was carried on a Freeze Dryer Lyophilizer VriTis Benchtop 4KBTZL.

In order to evaluate drug loading efficiency of PL-DFX, calibration curve of DFX has been illustrated by the UV absorbance of 0, 2, 4, 6, 8, 10 µg/ml DFX dissolved in PBS. The absorbances of diluted solutions were measured at 245 nm using UV/Visible spectrophotometer. The plot of UV absorbance *versus* DFX concentration was subjected to linear regression analysis. The 1.00 mg of PL-DFX was weighed precisely, the amide bond linked polylysine and DFX was hydrolyzed by aqueous solution of HCl (2 M), the DFX was separated by chromatographic column and dissolved in 50 ml PBS to determine the drug concentration. The drug loading efficiency was calculated based on equation LE (%) = We/Wm × 100%.

### Cell culture

The human gastric carcinoma cell (HGC-27) was cultured in RPMI 1640 (Gibco). The human colorectal carcinoma cells (DLD-1 and HCT-116) were cultured in RPMI 1640 (Gibco) and McCoy’s 5A respectively. The human renal tubular epithelial cell (HK2) was cultured in DMEM (Gibco). All media were supplemented with 10% fetal bovine serum (FBS, Gibco) and 1% penicillin-streptomycin (Gibco). All cells were cultured and incubated in a humidified atmosphere at 37°C with 5% CO_2_.

### Cell viability

Cell viability was analyzed by cell-counting kit-8 (CCK8) assay. Briefly, cells were seeded into 96-well plates at a density of 5000–10000 cells per well and incubated overnight. Then drugs were added at different concentrations. 48 h after treatment, cell viability was measured according to the manufacturer’s instructions.

### Apoptosis assay

Evaluation of apoptosis assay was performed by using Apoptosis Kit. According to the manufacturer’s instructions, cells were seeded into 6-well plates and incubated with PL-DFX (25 μM) at 37°C for 0 h, 24 h and 48 h. Then the cells were collected and stained with Annexin V-FITC and PI for 20 min. After staining, the cells were evaluated by flow cytometry.

### Wound healing

HGC-27 cells were seeded into 6-well plates at the density of 5×10^5^ per well and incubated for 24 h at 37°C. Then using a sterile pipette tip to scratch the cells. After washing 3 times with PBS, the medium containing DFX (12.5 μM) and PL-DFX (12.5 μM) was added to the wells. Finally, photos of wound healing were taken at 0 h and 24 h, respectively.

### Transwell

Transwell chambers were placed into a 24-well plate and 60 μl of diluted Matrigel was added to each chamber. Once the Matrigel was solidified at 37°C, 200 μl of cell suspension (5×10^4^ cells per chamber) which contained DFX (40 μM) and PL-DFX (40 μM) was added into the chamber, and 700 μl of medium containing 10% FBS in the lower chamber. Then cells were incubated at 37°C for 48 h. After washed two times with PBS, cells were fixed with 4% paraformaldehyde for 30 min and dyed with crystal violet for 30 min. Removed the excess dye, the chamber were dried at room temperature and photos were taken.

### Labile iron pool

The cellular LIP was measured as described previously (Prus and Fibach, 2008; Komoto et al., 2021). Briefly, cells were seeded into 6-well plates and incubated with DFX (25 μM) and PL-DFX (12.5 μM) for 48 h. After washed with PBS, these cells were incubated with calcein-AM (0.5 μM) at 37°C for 15 min protected from light. The mean fluorescence intensity was measured by using flow cytometry.

### Cell cycle

Cells were seeded into 6-well plates and cultured at 37°C overnight. Then cells were incubated with DFX (25 μM) and PL-DFX (12.5 μM) for 24 h. At the end of incubation, cells were collected and fixed in 75% ethanol for 24 h at 4°C. Then the cells were washed with PBS and stained with propidium iodide (PI) solution containing RNase A at 37°C for 30 min protected from the light. Finally, the cells were analyzed by flow cytometry.

### Mitochondria membrane potential

Cells were seeded into 6-well plates and incubated overnight. Then cells were treated with DFX (50 μM) and PL-DFX (25 μM) for 24 h. After treatment, cells were stained with JC-1 dyeing working solution for 20 min and washed three times with JC-1 buffer according to the manufacturer’s protocol. Finally, cells were collected and analyzed by flow cytometry.

### Establishing and passaging of organoids

This study was approved by the ethical committee of the Seventh Affiliated Hospital of Sun Yat-Sen University and performed in compliance with the Declaration of Helsinki. Written informed consents were obtained from all patients.

### Establishing of organoids

The clinical specimens were rinsed in PBS containing penicillin-streptomycin 5 times and then sheared into 1–3 mm^3^ small piece. The tissue fragments were digested for 2 h and supernatant was taken. After centrifugation, supernatant was removed and the remaining was resuspended with DMEM containing FBS. Centrifuged again, the cells were resuspended with DMEM and Matrigel at the ratio of 1:1 by volume. The mixed liquid was added into prewarmed 96-well plates with 10 μl per well then incubated at 37°C for 30 min. Once the Matrigel was solidified, 100 μl of medium was added to each well and cells were cultured at 37°C with 5% CO_2_.

### Passaging of organoids

Organoids were digested with TripLE (Gibco) at 37°C for 30 min and then centrifuged at 7000 rpm for 1min. The supernatant was discarded and cells were washed one time with DMEM. Then cells were resuspended in DMEM and mixed with Matrigel (Corning) at the ratio of 1:1 by volume. Subsequent steps were described above.

### Cytotoxicity of PL-DFX in organoids

Organoids were digested, centrifuged and resuspended as previously described. The cell suspension was added into prewarmed 96-well plates with 5 μl per well and then incubated at 37°C for 30 min. Once the Matrigel was solidified, 75 μl of organoid-conditioned medium was added and cells were cultured at 37°C and 5% CO_2_. Two days later, different concentrations of PL-DFX (75 μl) were added to the wells and organoids were cultured for an additional 120 h. Then, 10 μl of CCK8 reagent was added into each well and incubated for another 4–6 h, and the absorbance of each well was measured at 450 nm by microplate reader (BioTek, SynergyH1, United States).

### Statistical analysis

Data was analyzed by GraphPad 8, and results were presented as mean ± SD. Comparisons between two independent groups were performed by using Student’s *t* test. *, ** and *** indicate that *p-value* < 0.05, 0.01 and 0.001, respectively.

## Results and discussion

### Design, preparation, and characterization of PL-DFX

**FIGURE 2:**
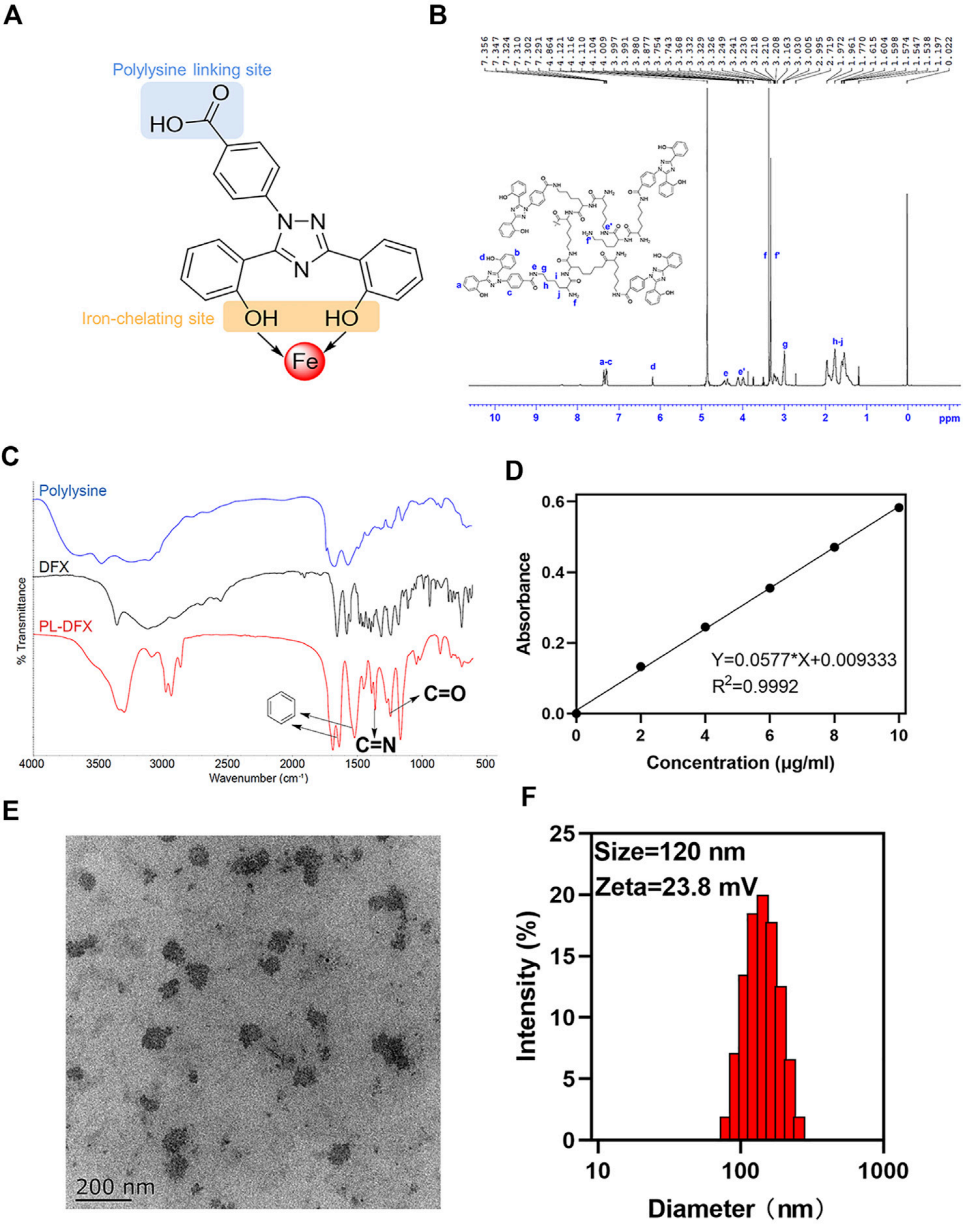
Characterization of PL-DFX nanochelator. **(A)** The polylysine linking site and iron-chelating site in the structure of DFX. **(B)** 1H-NMR spectra of PL-DFX. **(C)** FTIR spectra of PL-DFX. **(D)** UV absorbance *versus* DFX concentration plot. **(E)** TEM image of PL-DFX. **(F)** Size distribution of PL-DFX.

The iron nanochelator PL-DFX composed of deferasirox and hyperbranched polylysine. In the choice of DFX and polylysine linkage site, we have carefully selected the carboxyl group on the opposite site to the“iron-catching”domain of DFX in order to avoid its iron decrease potency (Figure 2A). Firstly, the hyperbranched polylysine was synthesized, followed by DFX loading *via* amide bond formation based on the protocol in the method part. The purified product was characterized by ^1^H NMR (Bruker 600 MHz. MeOD, 298K) and FTIR (Figures 2B,C). According to the FTIR spectrum of PL-DFX, peaks at 3296.88 cm^−1^ represented the O-H streching, and the peaks at 1643.38 and 1625.29 cm^−1^ represented the benzene streching in the DFX molecule. Additionally, the multi peaks at 7.291–7.356 ppm of ^1^H NMR spectrum belong to the aromatic ring proton of DFX, which indicated the drug loading was successful.

Moreover, according to the UV absorbance of DFX (Figure 2D), the drug loading efficiency calculated was 34%. Size and zeta potential are key parameters of nanoparticle efficacy. Nanoparticle with around 100 nm diameter and positive charge can be more easily uptaken by tumor cells (Albanese et al., 2010). The transmission electron microscopy (TEM) demonstrated that PL-DFX was spherical in morphology and monodisperse nanoparticles (Figure 2E). The diameter and zeta potential of PL-DFX, as illustrated by Figure 2F, were 120 nm and 23.8 mV, respectively.

### Cell viability and apoptosis

**FIGURE 3:**
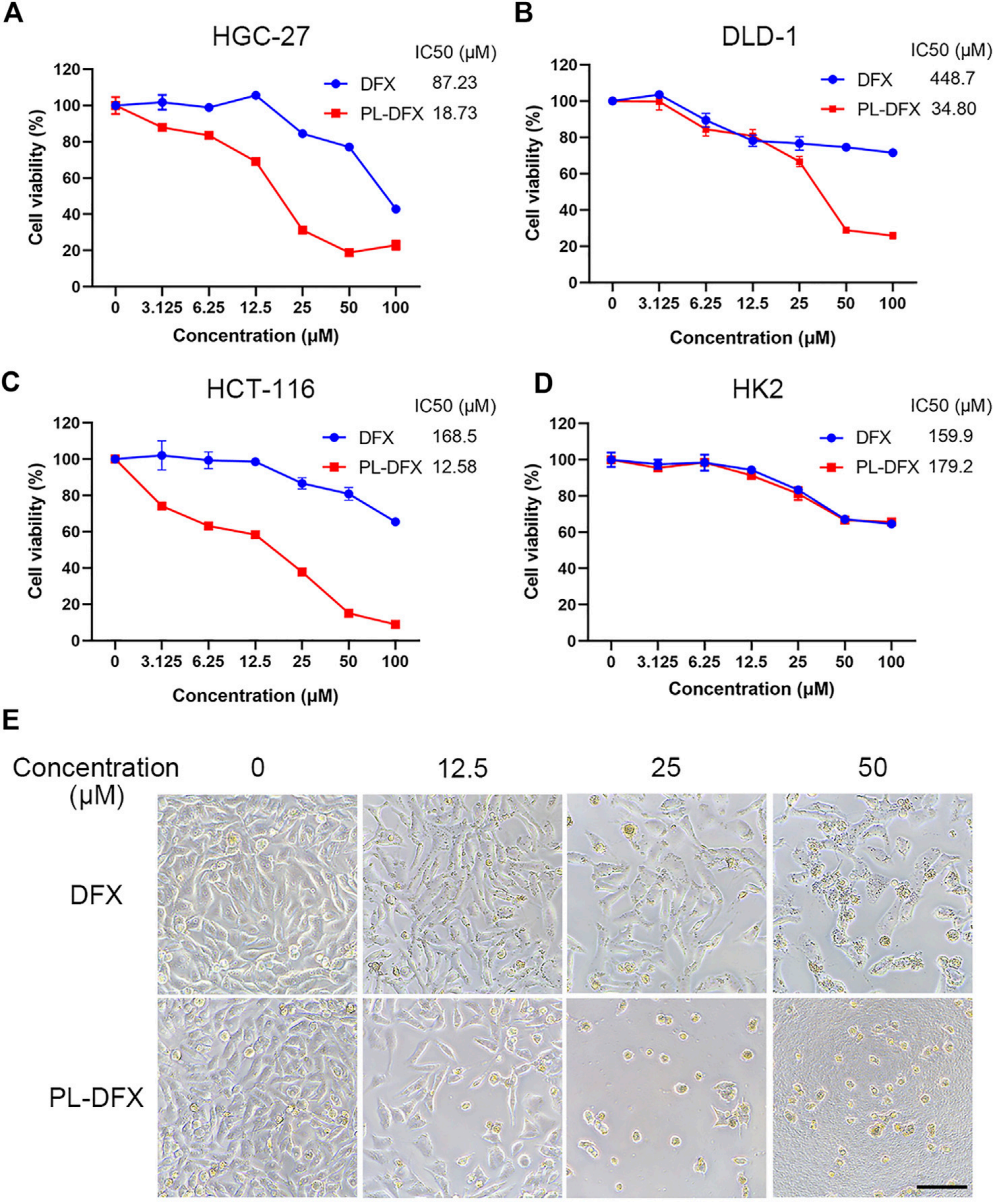
Cytotoxicity of PL-DFX *in vitro*. **(A–C)** Cell viabilities of HGC-27, DLD-1, and HCT-116 after incubated with DFX and PL-DFX for 48 h was measured by CCK8. **(D)** Cell viabilities of HK2 after incubated with DFX and PL-DFX for 24 h was measured by CCK8. **(E)** Microscopic images of HGC-27 after incubated with DFX and PL-DFX for 48 h. Scale bar = 100 μm.

Accumulating evidence has confirmed the antitumor effects of DFX *in vitro* and *in vivo* (Ford et al., 2013; Amano et al., 2020; Zhou et al., 2022). Polylysine-based delivery platform can enhance the anti-tumor effects of drugs (Thambi et al., 2016; Toshiyama et al., 2019). Herein, CCK8 assay was used to evaluate the cytotoxicity of DFX and PL-DFX in HGC-27, DLD-1, and HCT-116 cells. IC50 value was also calculated from the dose-response curves shown. As illustrated in Figures 3A–C, cytotoxicity of DFX and PL-DFX against these tumor cells was in a concentration-dependent manner, and cell viability decreased with increasing concentrations of drugs. Compared to DFX, cells incubated with the same concentration of PL-DFX (at equivalent concentrations of DFX) showed lower viability. PL-DFX inhibited HGC-27, DLD-1 and HCT-116 with IC50 values of 18.73 μM, 34.80 μM and 12.58 μM, which were significantly lower than IC50 values of DFX (87.23 μM, 448.7 μM and 168.5 μM, respectively). The bright field images also showed that PL-DFX exhibited greater ability to inhibit cell proliferation than DFX (Figure 3E). The higher the PL-DFX concentration, the lesser number of cells, and the cells became small and round.

Polylysine, as a biodegradable drug delivery platform, not only enhances the cytotoxicity of free drugs in tumor cells, while showing no higher toxicity in non-tumor cells (Du et al., 2020; Zhu et al., 2021). Due to nephrotoxicity of DFX, the cytotoxicity of DFX and PL-DFX was also investigated in human renal tubular epithelial cells (HK2). PL-DFX did not display enhanced cytotoxicity in HK2 cells, which was similar with DFX (Figure 3D). These results confirmed that the antitumor effect and biosafety of PL-DFX were superior to DFX.

**FIGURE 4:**
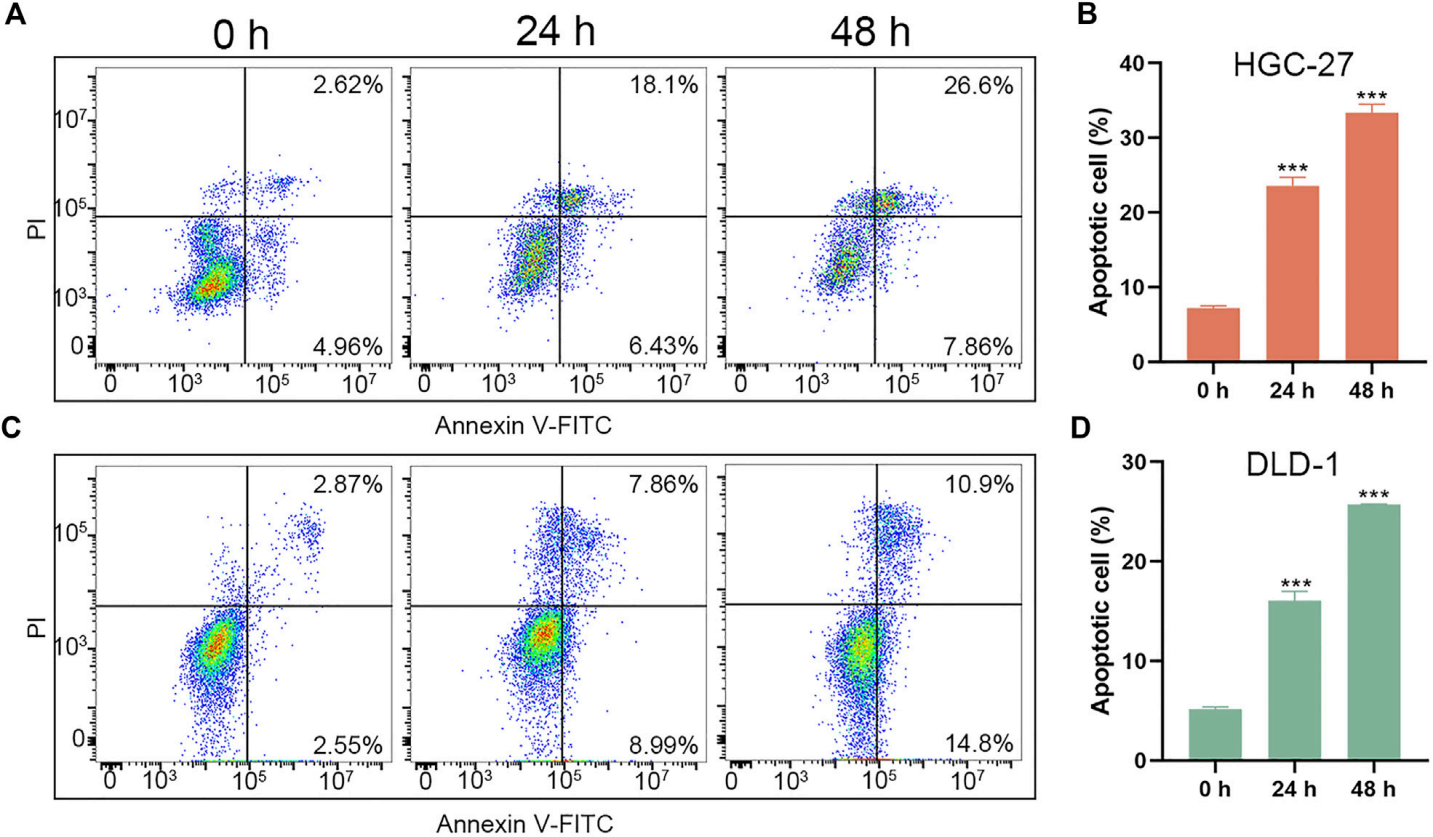
Pro-apoptotic effect of PL-DFX in HGC-27 and DLD-1 cells. **(A,B)** Apoptotic cells rate of HGC-27 incubated with PL-DFX (25 μM) for 0 h, 24 h and 48 h **(C,D)** Apoptotic cells rate of DLD-1 incubated with PL-DFX (25 μM) for 0 h, 24 h and 48 h.

Besides, we further evaluated the pro-apoptotic effects of PL-DFX against HGC-27 and DLD-1 by using Annexin V-FITC/PI kit. As illustrated in Figures 4A–D, the percentage of overall apoptotic cells incubated with PL-DFX for 48 h was 34.46% in HGC-27 and 25.7% in DLD-1, which was significantly higher than that at 24 h (24.53% in HGC-27 and 16.85% in DLD-1) and 0 h (7.58% in HGC-27 and 5.42% in DLD-1). These results indicated the time-dependent cytotoxicity of PL-DFX. In summary, polylysine carrier could enhance the anti-tumor effects of deferasirox in the tumor cells.

### Migration and invasion

**FIGURE 5:**
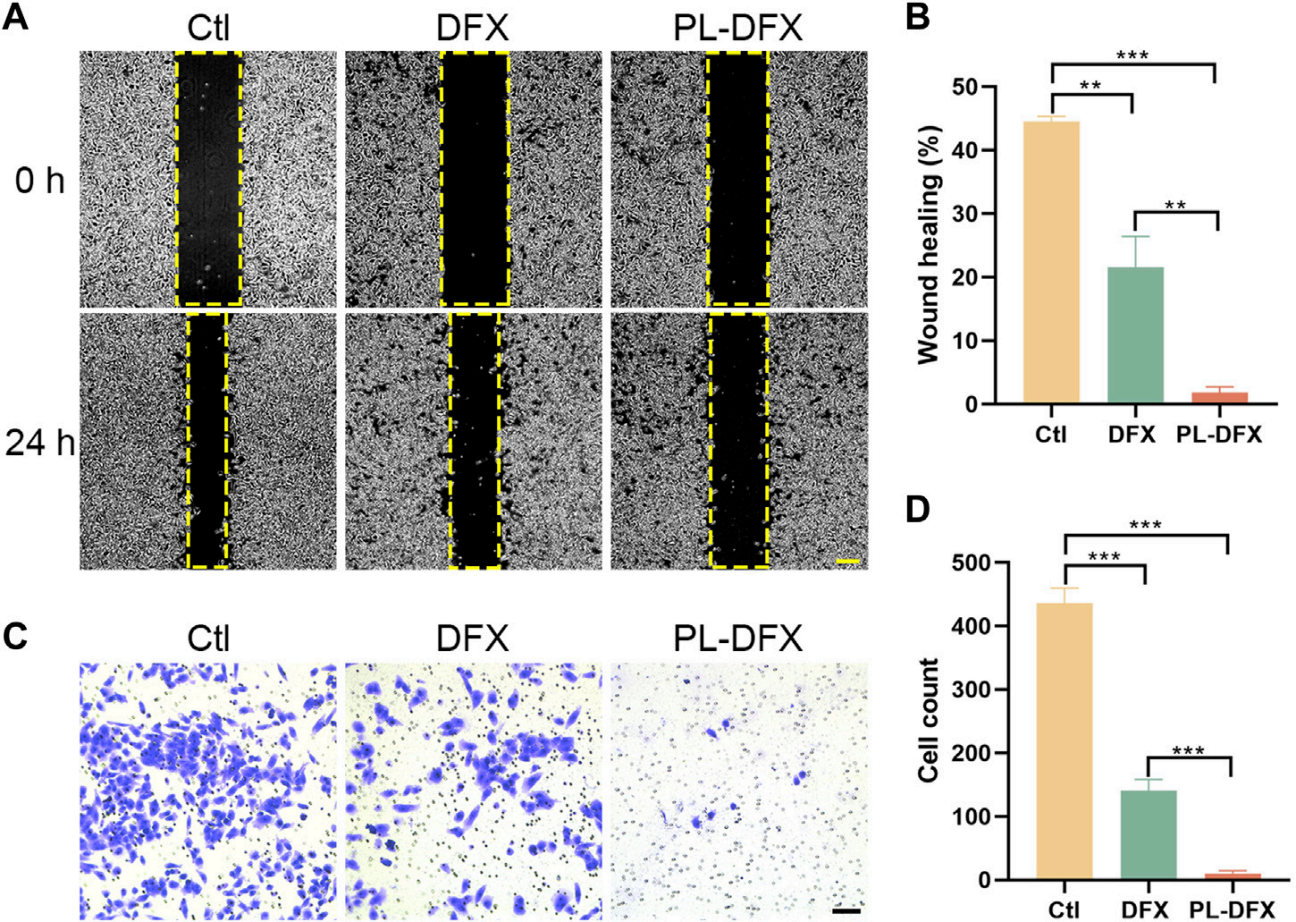
Inhibition of migration and invasion *in vitro*. **(A,B)** Microscopic image and statistical chart of wound healing assay in HGC-27 incubated with DFX (12.5 μM) and PL-DFX (12.5 μM) for 24 h. Scale bar: 200 μm. **(C,D)** Microscopic image and statistical chart of transwell invasion assay in HGC-27 incubated with DFX (40 μM) and PL-DFX (40 μM) for 48 h. Scale bar: 100 μm.

Next, we also explored the impact of DFX and PL-DFX on cell migration and invasion *in vitro* by wound healing and transwell invasion assays. As shown in Figures 5A,B, after 24 h incubation, the wound healing rate of PL-DFX group was 2.53%, which was significantly lower than that of control and DFX groups (43.65% and 25.66%, respectively). The transwell assay also displayed that the cell count of invasive HGC-27 cells incubated with PL-DFX for 24 h was significantly lower than that of control and DFX groups (Figures 5C,D). The above observation indicated that the ability of DFX loaded on polylysine to inhibit migration and invasion was greater than free deferasirox.

### Disruption of iron metabolism

**FIGURE 6:**
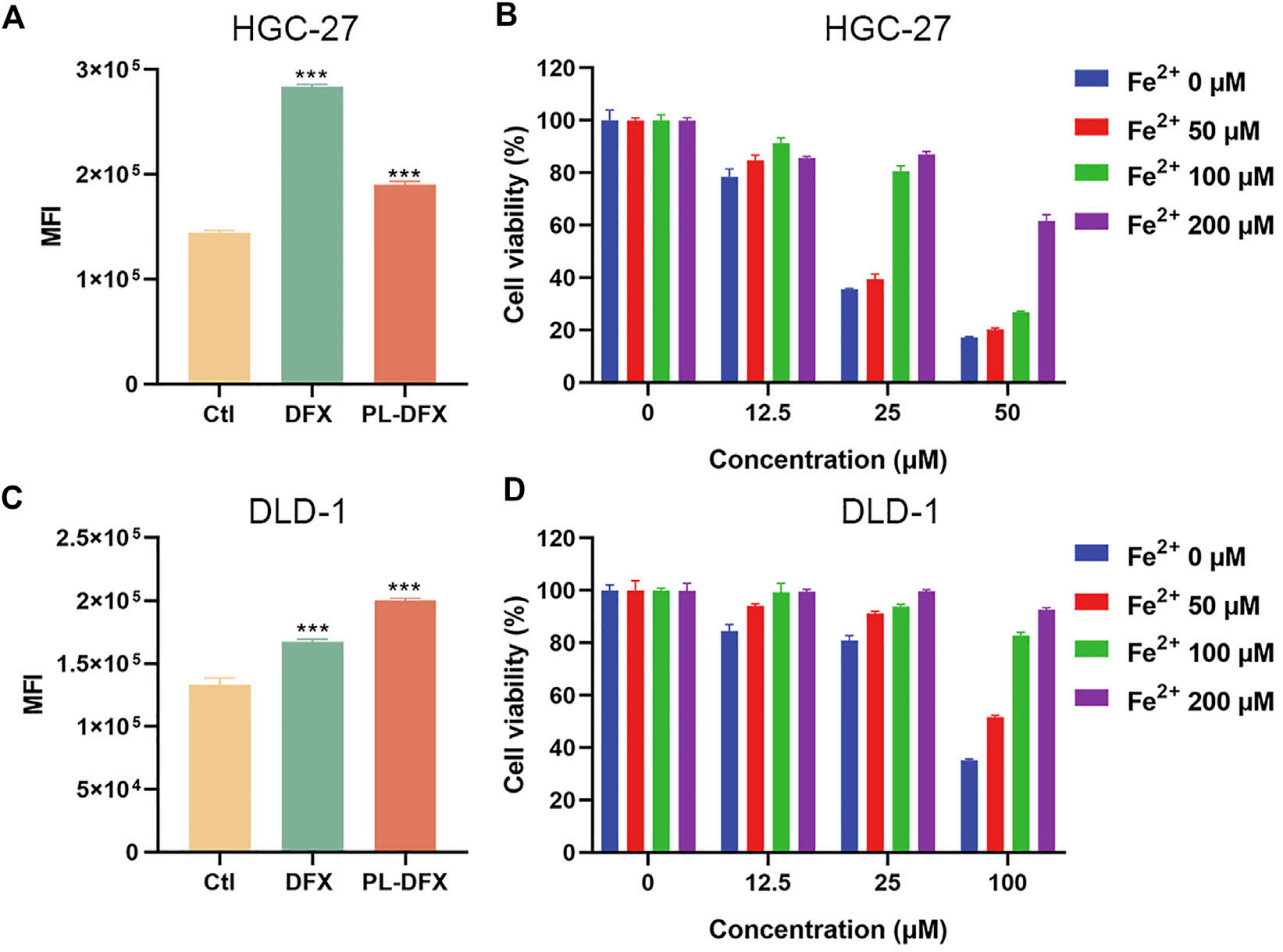
Disruption of cellular iron metabolism. **(A,C)** Flow cytometric analysis of intracellular iron of HGC-27 and DLD-1 using calcein-AM after treatment with DFX (25 μM) and PL-DFX (12.5 μM) for 48 h **(B,D)** Cell viabilities of HGC-27 and DLD-1 after treatment with PL-DFX in the presence or absence of Fe^2+^ for 48 h.

The cellular labile iron pool (LIP) was measured by using the calcein-AM (Prus and Fibach, 2008; Komoto et al., 2021). Although, calcein-AM has been frequently used to evaluate the cell viability and calcium, its fluorescence is quenched when binding with cellular iron. Iron chelators can inhibit the formation of complexation and increase the fluorescence intensity of calcein. Therefore, the changes in fluorescence intensity of calcein indicates the change in intracellular iron levels. As shown in Figures 6A,C, compared with the control, HGC-27 and DLD-1 cells incubated with DFX and PL-DFX had increased calcein fluorescence, indicating reduction of intracellular iron levels and similar iron-chelating ability of both. Moreover, after incubation with PL-DFX, supplement of Fe^2+^ can partially reverse the cytotoxicity of PL-DFX in HGC-27 and DLD-1 cells (Figures 6B,D). Herein, in the structure of PL-DFX nanoparticles, the conjugation between polylysine and deferasirox do not affect iron chelating ability of deferasirox.

### Cell cycle and mitochondrial membrane potential

**FIGURE 7:**
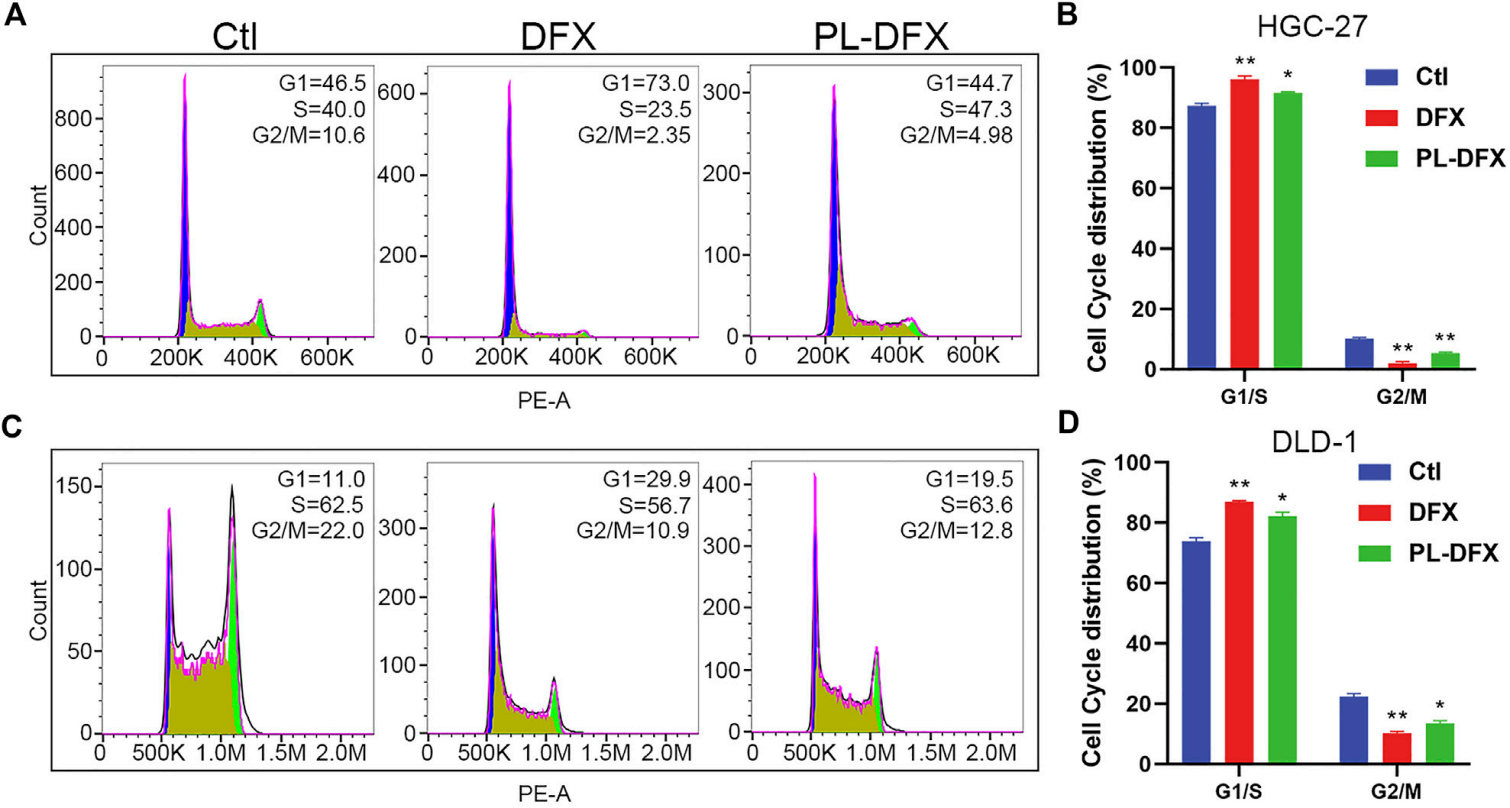
Impact of DFX and PL-DFX on cell cycle. **(A,B)** Cell cycle distribution of HGC-27 incubated with DFX (25 μM) and PL-DFX (12.5 μM) for 24 h **(C,D)** Cell cycle distribution of DLD-1 incubated with DFX (25 μM) and PL-DFX (12.5 μM) for 24 h.

We have demonstrated that PL-DFX has similar iron chelating ability with DFX. Next, the impact of PL-DFX on biological functions of HGC-27 and DLD-1 cells was performed. Iron is essential for the activity of ribonucleotide reductase, and iron depletion can inhibit the DNA synthesis and cause G1/S arrest (Chantrel-Groussard et al., 2006; Yu et al., 2007). Herein, as shown in Figure 7, similar to the previous study, compared to the control group, cells incubated with DFX displayed obviously phase G1/S arrest. As expected, PL-DFX also resulted in an increased proportion of cells in G1/S phase.

**FIGURE 8:**
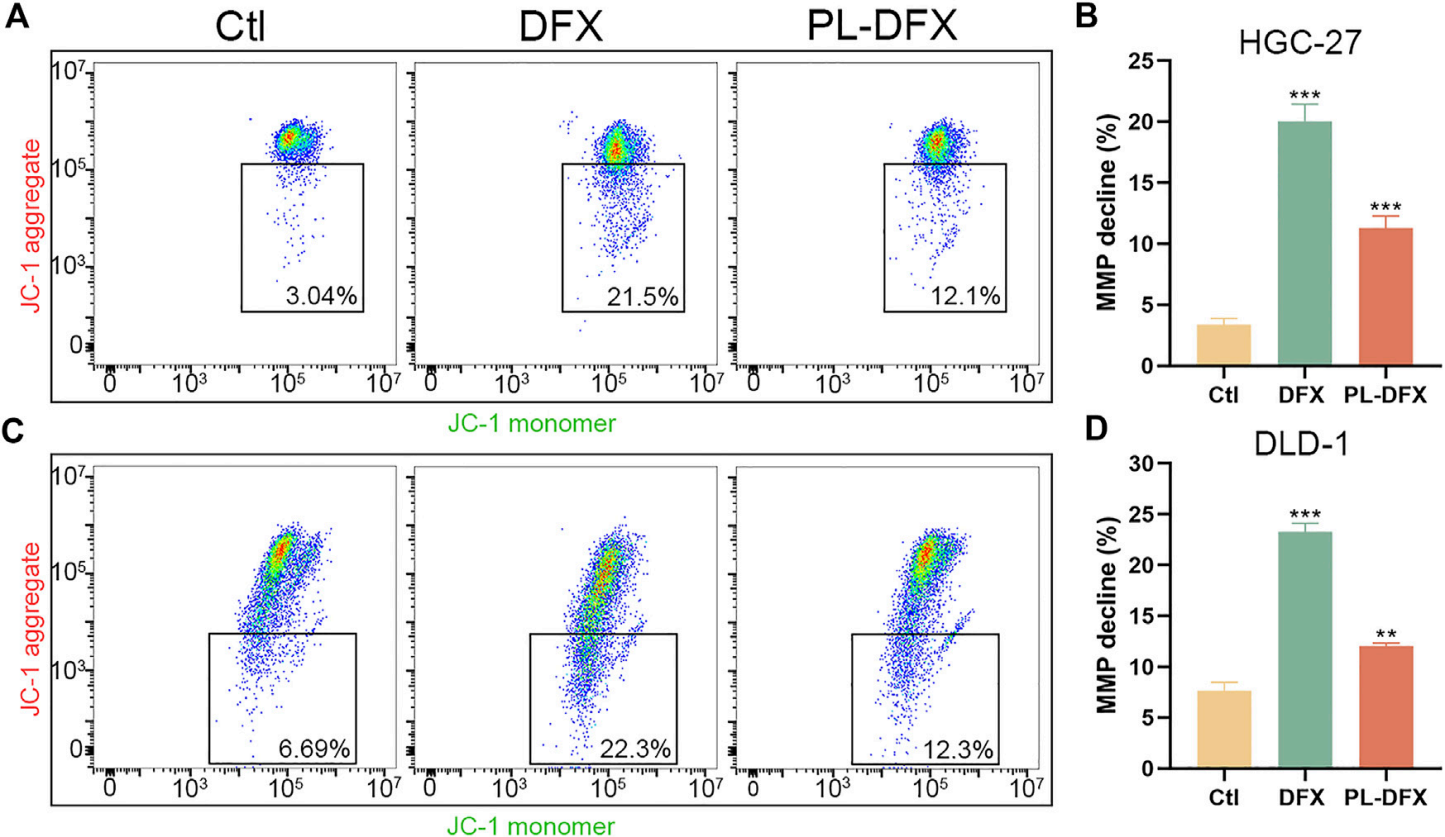
Impact of DFX and PL-DFX on mitochondria membrane potential. **(A,B)** Mitochondria membrane potential detected by JC-1 in HGC-27 cells incubated with DFX (50 μM) and PL-DFX (25 μM) for 24 h **(C,D)** Mitochondria membrane potential detected by JC-1 in DLD-1 cells incubated with DFX (50 μM) and PL-DFX (25 μM) for 24 h.

Iron plays a key role in mitochondria biological function and biosynthesis (Paul et al., 2017), and iron deficiency can compromise mitochondria function (Hoes et al., 2018). Decreased mitochondria membrane potential (MMP) is a hallmark of mitochondria dysfunction. Thus, the impact of DFX and PL-DFX on mitochondria membrane potential was investigated by using JC-1. As illustrated in Figure 8, iron deficiency induced by DFX resulted in decreased MMP. Similar to DFX, PL-DFX also depolarized the MMP in HGC-27 and DLD-1 cells.

### Cytotoxicity of PL-DFX in organoids

**FIGURE 9:**
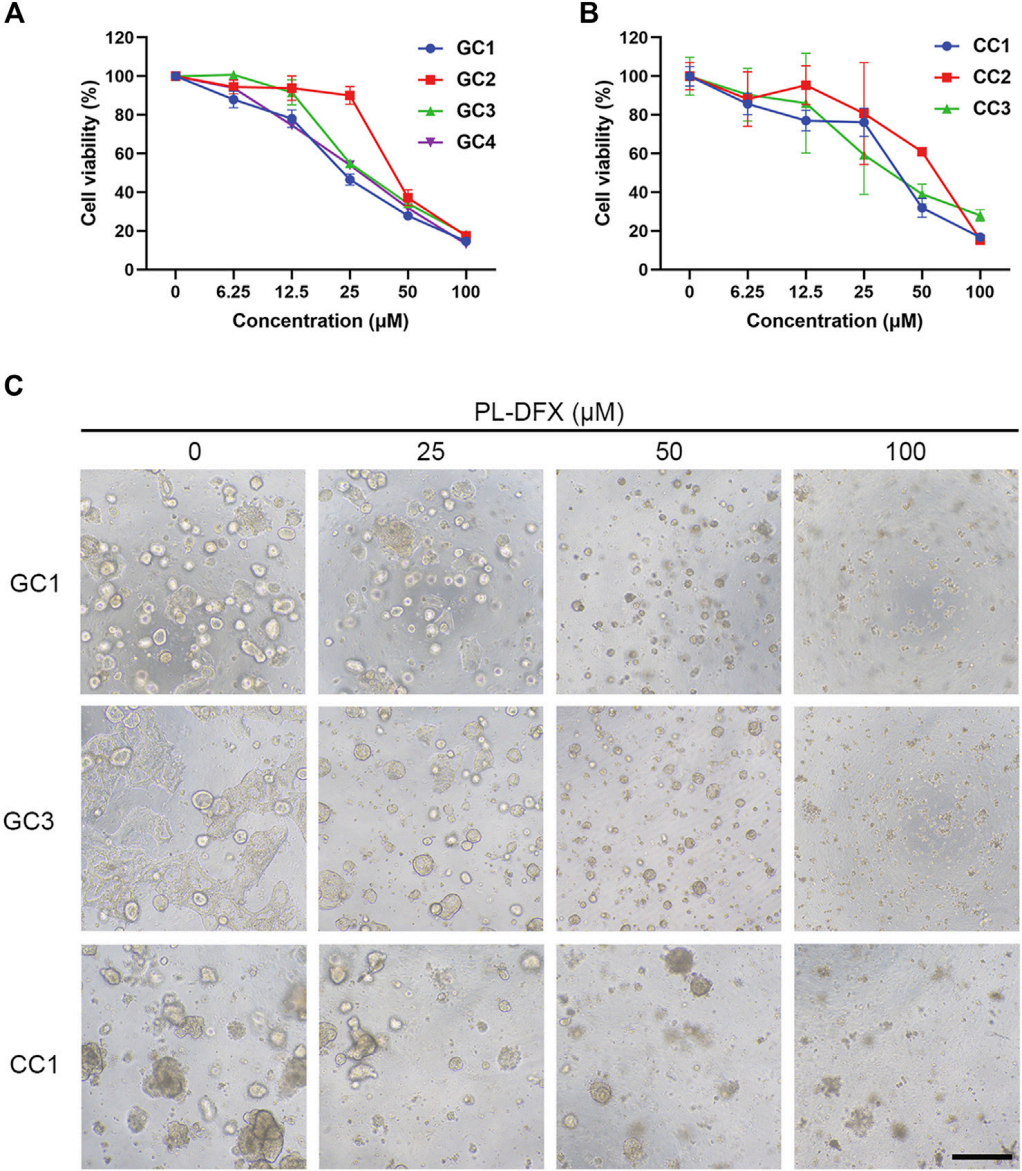
Cytotoxicity of PL-DFX in organoids. **(A)** Cell viability of gastric cancer organoids after treatment with various concentrations of PL-DFX for 120 h. **(B)** Cell viability of colorectal cancer organoids after treatment with various concentrations of PL-DFX for 120 h. **(C)** Microscopic images of gastric and colorectal cancer organoids after treatment with PL-DFX for 120 h. Scale bar: 300 μm. (GC: gastric cancer organoid; CC: colorectal cancer organoid).

Cell line is an important platform for drug development and screening. However, variations between cell lines and original tumors are responsible for the failure of the drug-based clinical trials (Kamb, 2005; Caponigro and Sellers, 2011; Barretina et al., 2012). Organoid, which established from patient-derived tumor tissue, resembles the original tumors in terms of biological characteristics and heterogeneity, and organoid-based drug screening methods have yield satisfactory results (Drost and Clevers, 2018). Therefore, we utilized the organoids which established from patient-derived gastric and colorectal cancer tissues to evaluate the clinical efficacy of PL-DFX. As shown in Figures 9A,B, the viability of organoids decreased with increasing the concentrations of PL-DFX. Besides, the PL-DFX treated organoids displayed smaller in size and fewer in number in compared to control organoids in both gastric cancer organoids (GC) and colorectal cancer organoids (CC) (Figure 9C).

## Conclusion

In summary, a novel iron nanochelator (PL-DFX) was designed and successfully synthesized *via* conjugation chemically between polylysine and deferasirox. This iron nanochelator, which prepared with around 120-nm diameter and positive charge, displayed higher cytotoxicity and greater capacity to inhibit migration and invasion than free DFX. Similar to DFX, PL-DFX also could disrupt cellular iron metabolism and biological functions involved iron, such as cell cycle and mitochondria membrane potential. Besides, its efficacy was also validated in the gastric and colorectal tumor organoids. Taken together, this study provides a new insight for cancer therapy *via* iron chelation.

## Data Availability

The raw data supporting the conclusions of this article will be made available by the authors, without undue reservation.
